# Point-of-care testing—new horizons for cross-sectional technologies and decentralized application strategies

**DOI:** 10.1007/s00216-022-03987-8

**Published:** 2022-03-10

**Authors:** Oliver Hayden, Peter B. Luppa, Junhong Min

**Affiliations:** 1 Heinz-Nixdorf-Chair of Biomedical Electronics, Department of Electrical and Computer Engineering, TranslaTUM, Campus Klinikum rechts der Isar, Bau 522, Einsteinstraße 25, 81675 München, Germany; 2 Institute of Clinical Chemistry and Pathobiochemistry, Klinikum Rechts Der Isar, TU München, Ismaninger Str. 22, 81675 München, Germany; 3 Department of Biomedical Engineering, School of Integrative Engineering, Chung-Ang University, 84 Heukseok-ro Dongak-gu, 06974 Seoul, South Korea

Near-patient biochemical testing, also called point-of-care testing (POCT), enables healthcare professionals and caregivers to perform rapid clinical laboratory testing in close proximity to the patient or directly at the patient’s side. POCT also allows patients to perform self-monitoring in chronic disease states. In both cases, predictive biomarkers for clinical decision-making or self-management of for instance diabetes or antithrombotic therapies are of pivotal importance. Because delays are no longer caused by clinical sample preparation, transport, or central laboratory analysis, innumerable clinical applications are possible that shorten the clinical decision-making time with implications for additional testing or therapy. Even though the field of omics increases our understanding of cellular mechanisms exponentially, only a few new diagnostic biomarkers were identified in the last two decades with clinical impact usually due to a lack of therapy options. On the other hand, new biomarkers can be important drivers for therapeutic research and preventive medicine.

POCT has been seen as a disruptive technology already for several years. Novel transducer technologies, miniaturization, multiplexing, and smartphone-derived IT tools nowadays enable test results to be viewed within minutes. Thus, clinical chemistry analysis moves away from centralized laboratories to near-patient diagnostics, whenever there is an advantage for clinical decision-making. In particular, for patients suffering from chronic disease or at the intensive-care unit, POCT is an opportunity for personalization of treatments. Here, continuous monitoring modality is the noble clinical goal for the near future! Already today, continuous glucose monitoring (CGM) is widespread and beneficial in the millionfold use.

The analytical approaches, described in three papers of this topical collection (O´Connell, Tombelli and Lefter) and linked to this topic, go far beyond the one-dimensional measurements of interstitial glucose concentrations that today’s CGM devices are able to perform. An easy-to-install permanent intravascular microdialysis catheter is the key to a continuous measuring mode! O’Connell and Krejci describe the theoretical background and capabilities of microdialysis techniques in biochemical analytics. In vivo measurements, performed by the use of microdialysis catheters, are subject to dynamic changes due to mass transfer and provide valuable information about the patient’s physiological state. Tombelli et al. offer a fascinating view of a future continuous monitoring of therapeutic drug (TDM) blood levels. TDM–related immunoassays are performed at the surface of a sophisticated disposable plastic chip in conjunction with bioconjugated submicrometric fluorescent magnetic particles. And finally, Lefter et al. presents a novel, tunable electrochemical sensor for wearable continuous, multi-metabolite monitoring. The multi-analyte sensing platform utilizes gold and platinum surfaces allowing fully reversible and stable sensing of different redox species in solution with superior analyte selectivity.

In addition to continuous measurements, POCT has the potential to support acute care diagnostics. Electrochemical sensing was employed by Yagati et al. to detect thrombin in clinical samples directly. The indium tin oxide (ITO) electrode with polypyrrole and palladium was synthesized, and liposomes encapsulating K_4_[Fe (CN)_6_] as labeling agent were introduced to maximize the signal corresponding to thrombin from a human serum sample. This aptamer-based electrochemical biosensor showed quantitative measurement for the linear segment (dynamic range, 0.1–1000 nM) of thrombin in serum with an LOQ of 1.1 pM and an LOD of 0.3 pM.

Less acute but of relevance for newborn development are the oligosaccharide diagnostics of breast milk discussed by Chung et al. Using impedimetric biosensing, a handheld device is reported, which allows determining the content of 2′-fucosyllactose with implications for infant health and development. The authors correlated impressively the lectin sensor accuracy for 2′-fucosyllactose with HPLC using banked milk samples for validation in a blinded study. Increased analytical sensitivity and new materials for reagents are not only necessary for electrochemical sensing tools but also important topics for novel lateral flow assay (LFA)–type platforms. Rink et al. discussed the commercially available fine-tuned liposomes (350 nm) containing sulforhodamine B (SRB) to detect interleukin 6 (IL-6) from human serum using the photometric LFA format. The newly designed liposomes with a high encapsulating load (150 mmol L^−1^ SRB) easily outperformed normal gold nanoparticles in photometric LFAs (LOD 7 pg mL^−1^) with an extraordinary shelf life of over 1 year at ambient temperature and up to 8 years under a liquid-phase storage condition.

The analytical sensitivity could be also dramatically increased by combining LFA with nucleic acid amplification methods. Agarwal et al. focused loop mediated isothermal amplification (LAMP) combined with LFA–formatted POCT for SARS-CoV-2 N-gene detection in clinical swab–extracted RNA samples. The LAMP protocol was newly designed with the biotinylated dUTP and FITC–labeled primer to incorporate in a lateral flow strip sensor (control line with anti-IgG and test line with streptavidin) with an anti-FITC antibody–coated gold nanoparticle. The authors validated this isothermal nucleic acid amplification–combined POC LFA with 82 SARS-CoV-2–positive RNA samples and showed impressive accuracy of 81.66% with an LOD as low as *C*_t_ of 33 in RT-PCR. With cerium oxide nanoparticles which possess outstanding oxidation activities for organic molecules, Kong et al. achieved a simple yet elegant colorimetic C-reactive protein LFA without requiring an oxidizing agent or a reader with an LOD ~ 100 ng mL^−1^ in human serum. This platform idea with a robust reagent solution could increase the shelf life of LFA as it does not depend on H_2_O_2_ storage and a turn-around-time of 3 min was reported, which outperformed conventional LFA with an Au-NP readout.

Instead, advanced Au nanostructures used by Kim et al. show improved surface-enhanced Raman spectroscopy (SERS) conditions to detect C-reactive protein (CRP). SERS probes were synthesized with target-specific DNA complex conjugated with porous gold nanoplates (pAuNPs) consisting of methylene blue (as labeling probe) and target-specific aptamer-coated Au-Te nanostructures (as capture probe) on ITO substrates. With this nanomaterial-supported biosensing approach, a CRP LOD of 3.11 pM is reported in diluted clinical serum samples. By advancing the LFA format to the third dimension, a microfluidic-based biosensing approach was developed for a pump-free workflow integration and still accurate biomarker measurements.

Lee et al. proposed a three-dimensional paper-based microfluidic analytical device (3D-μPAD) capable of sophisticated multi-step reactions required for ELISA. The 3D-μPAD sensor was used to detect the breast cancer biomarker thioredoxin-1 in clinical human serum samples, and the precision of the device was validated with conventional ELISA. Despite integration, any progress for POCT also depends on the standardization of already-established measurements. There is a lot of catching up to do here. Consequently, DuBois et al. thankfully focus on their international study on the standardization of one of the oldest and still indispensable clinical chemistry parameter: creatinine in serum. This analyte allows the early identification of chronic kidney disease, a major global public health problem. Lastly, field testing of the POCT is required for clinical acceptance and a future innovation step, which was performed by clinicians around Wechselberger*.* With an optical immunoassay based on a smart microfluidic solution in close proximity to the photodetector, fast binding kinetics and high quantum yields are achieved, which allowed anti-SARS-CoV-2 IgG testing in the population of Austria and demonstrating POC opportunities for pandemic monitoring.

In the not-too-distant future, we expect to be able to check our individual health status with a simple act, such as checking the time. The perfect storm COVID-19 already demonstrates the impact of POCT to manage the countermeasures of societies. For hospital POCT, the notion of conventional and POCT laboratory services residing within the same health facility seems contradictory, but these two are, in fact, complementary. Together, POCT and the central laboratory are pivotal in covering the demands of diagnostic processes. This topical collection of ABC bears witness to the creative solutions this field offers.

